# [Retracted] MicroRNA‑29a inhibits cell migration and invasion by targeting Roundabout 1 in breast cancer cells

**DOI:** 10.3892/mmr.2023.13151

**Published:** 2023-12-18

**Authors:** Hui Li, Jiashun Luo, Bin Xu, Kaijun Luo, Juan Hou

Mol Med Rep 12: 3121–3126, 2015; DOI: 10.3892/mmr.2015.3749

Following the publication of this paper, it was drawn to the Editors’ attention by a concerned reader that the Transwell migration and invasion assay data shown in Fig. 3A and B were strikingly similar to data appearing in different form in other articles written by different authors at different research institutes that had either already been published elsewhere prior to the submission of this paper to *Molecular Medicine Reports*, or were under consideration for publication at around the same time (a few of which have already been retracted). In view of the fact that certain of these data had already apparently been published previously, the Editor of *Molecular Medicine Reports* has decided that this paper should be retracted from the Journal. The authors were asked for an explanation to account for these concerns, but the Editorial Office did not receive a reply. The Editor apologizes to the readership for any inconvenience caused.

